# Report of a Case of Rupture of the Male Urethra

**Published:** 1889-11

**Authors:** J. D. Thomas

**Affiliations:** Professor of Genito-Urinary and Venerial Diseases, Western Pa. Med, College


### REPORT OF A CASE OF RUPTURE OF THE MALE URETHRA.*

---

BY J. D. THOMAS, M. D., PROFESSOR OF GENITO-URINARY AND VENERIAL DISEASES, WESTERN PA. MED, COLLEGE.

---

The case I beg leave to report is one of rupture of the urethra, in its fixed portion, from trauma. These cases do not occur very often, but they are of sufficient frequency to merit attention. Like cases of strangulated hernia, if not interfered with surgically early enough, they almost inevitably result in the death of the patient. The text-books lay down certain rules of conduct to be followed, but I believe that a case from actual practice is more instructive, and often brings forward difficulties that the text-books may not touch upon; and, moreover, among gentlemen of much experience, such as compose the majority of

*Read at Allegheny County Medical Society.

the members of this society, the discussion may bring out points of very great interest and instruction.

J. S., aged 61 years, on April 3d, at 7 p. m., of the present year, whilst standing on a ladder with his back to the same, and in the act of stepping with one foot to the second floor of an outbuilding, accidentally slipped with the other foot that was upon the round of the ladder, and slid down backwards and struck with considerable momentum his perinæum upon a pick-handle that was resting against the ladder. When he struck upon the pick-handle he lunged forward, his body taking the handle with it for a moment, and then the handle projecting forwards between his thighs as he fell forward on his face. He felt considerably hurt, but did not become faint. After resting awhile, he visited a drug-store four blocks away and purchased a lotion for application.

At 10 p. m., or three hours after the accident, he made an attempt to pass his urine but failed, when he sent for me.

After getting the above history of the case I made an examination of the parts, and found the following conditions: There was already some discoloration of the perinæum, but no abrasion. On his under-drawers I found about a half-dram of blood, that had evidently come from the meatus. There was no distention of the bladder, as he had passed his water immediately before the accident. All I did at this visit was to give the patient opium, and order him to bed.

April 4, 8 a. m. The patient made an attempt to pass his urine, but failed. I then introduced a soft catheter, which passed to five inches, and after a little delay went one inch farther. On its removal there was a little blood in the eye. I then substituted an ordinary silver catheter, which would not pass. This also contained some blood in the eye. Both of these efforts were made with the utmost gentleness. At this visit there was some swelling and a good deal of discoloration of the perinæum and scrotum. Opium continued and sitz baths ordered—no attempt at catheterization.

3 p. m. Visited my patient prepared to aspirate if necessary, but he had urinated whilst in the bath some time before my arrival, and percussion over the pubes showed the bladder empty.

April 5, 8:30 a. m. The patient passed four ounces of urine during the night—this urine was saved for my inspection, and it contained no blood. He passed a restless night, however, owing to pain in the perinæum and scrotum. No distention of bladder. The scrotum is very large and almost black from extravasation of blood; the penis is now also discolored. Opium, and the scrotum supported.

3 p. m. Passed urine twice since morning visit, and feels comfortable, resulting, as he thinks, from the support given to the scrotum.

April 6, 9 a. m. Passed two pints of urine, at intervals of two hours, during the night. The scrotum is immensely distended, measuring sixteen inches in circumference; penis much swollen and discolored like the scrotum. Otherwise doing well.

April 7, 9 a. m. During the last 24 hours the patient passed a fair quantity of urine, somewhat discolored with blood. The penis is reduced in size, but scrotum the same as before.

April 8, 8 a. m. Two pints of urine passed since last visit. Patient complains sorely from the swelling in the scrotum.

11 p. m. Suffers very much from the distention of the scrotum. I passed a large aspirating needle in three or four places over the scrotum, expecting to find effused blood in the vaginal cavities, but nothing but a little bloody serum was evacuated.

April 9, 8 a. m. Passed a restless night. Some urine passed per urethram, but the discharge from the punctures made in the scrotum the day before has a urinous odor; this last fact caused me to decide at once as to the necessity of opening the urethra in the perinæum.

2:30 p. m. Operation. I attempted to pass solid instruments, in decreasing numbers, in order to have a guide, but failed. Fortunately, however, a soft instrument went past the obstruction, and this served the purpose. After cutting down upon the guide, a very free incision was made. On the right side of the urethra quite a slough was found. A soft catheter was then introduced into the bladder through the wound, and permitted to remain. Four incisions were made into the scrotum—two on each side of the raphe. The operation lasted little less than one hour, inclusive of anæsthesia.

April 10. Patient doing fairly well. Scrotum reduced to eleven inches in circumference. No fever.

April 11. Condition good. Evidence of a slough on the right side of the scrotum at the seat of one of the incisions. Made two more incisions on the right side.

April 12. Doing well. During the night the catheter became clogged, interfering with the discharge of urine. I removed the catheter, a silk one, and found it covered with incrustation, and the catheter-material disintegrating. Passed a soft rubber catheter per urethram and tied it in.

April 13. In fair condition. Catheter removed, but to be introduced every three hours, to insure complete emptying of the viscus. Poultices to be applied over the slough on the scrotum.

April 14. In splendid condition. All of the wounds looking well, including the sloughing one, which is cleaning off somewhat. Scrotum reduced to nearly normal.

April 15. Doing well.

April 16. Slough almost clean. Patient says he was a little chilly during the night, but no elevation of temperature has followed, and pulse is normal.

April 17. Doing well.

April 18. Doing well.

April 19. Slough entirely clean.

April 20. Wounds all doing well. Poultices discontinued.

April 21. Doing well.

April 22. Doing well.

April 23. Doing well.

Afiril 24. Doing well.

April 25. Doing well. Got out of bed to-day, and passed urine unaided for the first time.

April 26. Doing well. Catheter to be passed twice in the 24 hours, as there is about three ounces of residual urine.

April 28. Everything well.

April 30. Everything well.

May 2. Everything well.

May 4. Everything well.

May 6. All wounds on scrotum healed, and patient took a walk out of doors.

May 9. Doing all right.

May 20. Wound in perinæum healed soundly.

During the entire treatment the wounds were irrigated, frequently, with a solution of mercury bichloride 1:4000. After the perinæal section, the patient did not take a dose of medicine as all of the functions were performed normally, and there was at no time an elevation of temperature. A full-sized steel sound was passed every third day until the perinæal wound was perfectly healed, and occasionally afterwards. The catheter was discontinued about the time the wounds were all healed, as the patient emptied the bladder perfectly by his own efforts.

In this case I appreciated from the beginning the fact that the urethra was torn, and stood ready to open the perinæum at any time, but, as the notes show, the patient was voiding urine in sufficient quantities, and presented no dangerous symptoms. I presumed, therefore, that nature had glazed over the rent in the urethra, and that the patient would recover without a perinæal section. When I relieved the tension of the scrotum by multiple puncture (for this tension, I believe, was caused by capillary effusion), the urine was permitted to pass through the rent which had evidently not become glazed over, and then it passed through the loose scrotal tissues and gave the discharge from the punctures a urinous odor; for when the punctures were made there was no urinous odor to the fluid which was evacuated. There was, however, some infiltration in the neighborhood of the rupture, as the condition of the parts demonstrated at the operation.

We had in the case not only a rupture of the urethra, but of the deep perinæal fascia (known here as Buck's fascia) as well, for the urine passed directly to the scrotum without first showing evidences of infiltration behind this fascia.

I would not advise the opening of the perinæum in all cases simply because there was a rupture of the canal; but the moment infiltration of urine is suspected, operate immediately, and afterwards pass full-sized sounds until the healing of the wound has taken place.

Although this case could not have terminated better, an earlier operation, evidently, would have been proper.

